# Similar effect of running on visual and auditory time perception in the ranges of milliseconds and seconds

**DOI:** 10.3389/fpsyg.2023.1146675

**Published:** 2023-03-31

**Authors:** Irene Petrizzo, Eleonora Chelli, Tommaso Bartolini, Roberto Arrighi, Giovanni Anobile

**Affiliations:** Department of Neuroscience, Psychology, Pharmacology and Child Health, University of Florence, Florence, Italy

**Keywords:** time perception, motor control, self-motion, cross-modal perception, sub and suprasecond timing

## Abstract

**Introduction:**

The ability to accurately encode events’ duration is of critical importance for almost all everyday activities, yet numerous factors have been reported to robustly distort time perception. One of these is physical activity (i.e., running, walking) but, partly due to the variety of methodologies employed, a full comprehension of the role of exercise on the encoding of time has still to be achieved.

**Methods:**

Here we tackle the issue with a multifaceted approach by measuring the effect of vigorous running with a time generalization task for visual and auditory stimuli in the range of milliseconds (0.2–0.8 s) as well as seconds (1–4 s). At baseline, participants performed both the encoding and decoding at rest while in the experimental conditions the decoding was performed while running.

**Results:**

Our results indicate that physical activity in both duration ranges (sub-second and seconds) was expanded during running regardless of the sensory modality used to present the stimuli. Despite this generalized effect of running on perceived duration, we found evidence for the existence of independent timing mechanisms: (1) the perceptual biases induced by running in the two temporal regimes were uncorrelated, (2) sensory precision levels (Weber fraction) were higher for stimuli in the seconds range, (3) sensory precision levels were higher for auditory than for visual stimuli, but only within the sub-second range.

**Discussion:**

Overall, our results support previous findings suggesting (at least partially) separate timing mechanisms for short/long durations and for visual and auditory stimuli. However, they also indicate that physical activity affects all these temporal modules, suggesting a generalized interaction—via generalized and shared resources—between the motor system and the brain time mechanisms.

## 1. Introduction

Perceiving time is a pervasive activity, applying to most everyday tasks, spanning very different time scales (from milliseconds to several days) and involving all sensory modalities. Duration of sensory events can be passively perceived, however most of the interactions we have with the environment are not passive but characterized by active motor interactions. The relationship between duration perception and action is not unidirectional, and while time perception is of critical importance to keep synchronized motor routines, these, in turn, are capable to significantly influence the perception of duration. Numerous studies have been dedicated to this relationship in the past, but the reported evidence is not always consistent to each other. A previous study addressing the interplay between physical exercise and time perception was by Lambourne (2012) that asked participant while cycling or resting, to compare, in a same/different task, the duration of visual stimuli (lasting from 0.14 to 1.27 s) to a previously learned duration (generalization task). The results showed that, while cycling, perceived duration was expanded by about 15%, relative to time estimates made at rest. With a time reproduction task, Tonelli et al. (2022) replicated and expanded these results by showing that time distortions were still evident approximately 15-20 minutes after the end of physical activity. Moreover, despite time distortions which occurred for all the tested durations, they were stronger for stimuli within the milliseconds range (0.2-0.8 ms) compared to longer stimuli (1.6, 3.2 s). This long-lasting effect has been interpreted as a consequence of a dopaminergic or GABAergic modulation induced by physical activity, and the stronger effect for the milliseconds range as the consequence of a relatively higher involvement of motor control on this temporal range. Finally, in this study it was demonstrated that perceptual distortions induced by cycling did not generalize to all visual tasks as visual spatial estimates (distance between two stimuli) remained veridical to ruling out the role of general, a-specific factors.

The perceived dilation of time for visual stimuli in the milliseconds range induced by cycling has been recently generalized to another motor activity: treadmill running. By leveraging on the same methodology as Lambourne (2012) it was shown that running also provided a robust expansion of visual time in the millisecond range and that running, like cycling, selectively affected time perception by leaving non-temporal features such as numerosity unaffected (Petrizzo et al., 2022). However not all temporal distortions induced by running resemble those yielded by cycling. For example, while cycling distorts time perception for a long period after the end of the physical activity, the expansion of perceived time induced by running completely vanishes immediately after the end of the exercise. Indeed, the recalibration of the temporal mechanisms following the end of the exercise was so rapid that even in the first trials after the running phase, when for example heart rate and other physiological variables were still well above the baseline level, time estimates became veridical again (Petrizzo et al., 2022).

Beyond the milliseconds vs. seconds categorization, another common distinction in the timing literature is related to sensory modalities. It is now well established that, while vision dominates space perception over audition (Alais and Burr, 2004), audition largely dominates vision for time perception. For example, with an audio-visual temporal bisection task, Burr et al. (2009) demonstrated that visual timing can be “captured” by auditory stimuli, dragging visual time towards the auditory time to induce a “temporal ventriloquist” effect. The dominance of audition over vision on time perception has been related to the common finding that time sensory thresholds are much lower for auditory compared to visual stimuli, making auditory timing more reliable, at least in the millisecond range (for a review see Rammsayer, 2014). Clinical evidence also sustains this dissociation. For example, Tinelli et al. (2015) showed that auditory but not visual time sensory discrimination thresholds (in the millisecond range) are impaired in preterm children, a finding difficult to reconcile with the existence of single a-modal system. Importantly for the aim of the current study, the dominance of audition over vision is not constant across temporal ranges. The auditory modality has been shown to dominate sensory precision (lower thresholds) over the visual modality predominantly for stimuli within the millisecond range while similar thresholds have been reported for longer durations above the second (Rammsayer, 2014; Rammsayer et al., 2015). Overall, the current literature seems to suggest a (probably smooth) transition from a sensory specific timing mechanism to a more generalized system, as a function of stimuli duration.

Despite the literature suggesting different mechanisms for vision and audition, to the best of our knowledge only one study investigated the influence of physical activity on auditory time perception (Kroger-Costa et al., 2013). Interestingly, the results indicate that the effect of running on sound discrimination, in the range of milliseconds was task dependent: subjective time was found to be expanded when perceived time was measured with a discrimination task while no distortion was found when time was measured via a generalization task. In light of the literature discussed above, the interaction between action and time perception is indisputable, and seems to suggest a close relationship between the two system.

In line with this idea, back in 2003 Walsh (2003) advanced the possibility for the existence of an integrated system dedicated to the perception of time, space and quantity (likely to be located in the parietal lobe) aimed at making the interactions between the motor and the perceptual system efficient.

Crucially, previous studies have demonstrated that the influence of action on time perception is not limited to whole-body movements such as cycling and running. For example, Ayhan and Ozbagci (2020) asked participants to reproduce the duration of a previously seen moving visual stimuli (dot arrays lasting from 0 to 1.5 s). In an “active” visuo-motor condition, in each trial the duration of the to-be-reproduced stimulus was generated by the participants via a key press. In a “passive” condition, the same durations exploited in the active phase were used to define the duration of the visual stimuli that participants had to reproduce. The results demonstrate that perceived duration of self-generated intervals was compressed, compared to passive viewing. Similarly, Yokosaka et al. (2015) found that, during fast circular hand movements, visual duration was compressed relative to a resting condition. Evidence that perceived time can be expanded via self-produced motor routines have also been collected. Tomassini et al. (2018) found that perceived duration of visual stimuli was expanded when these were presented in the middle of two consecutive finger taps, while duration was compressed for stimuli displayed near tap onsets, to reveal a dynamic coupling between action and perception. Anobile et al. (2020a) showed that visual time can be distorted by a motor routine even when this has already ceased. In this experiment, participants performed mid-air tapping movements for a few seconds, either slowly or quickly (tested in separated sessions). Soon after the end of the motor phase they were asked to judge the relative duration of two drifting gratings, one spatially coincident with the tapped region and the other in a neutral location in the opposite visual field. The results revealed that, after fast tapping, perceived duration was compressed around the tapping region while slow tapping induced a perceived expansion of time.

To summarize, the literature consistently points toward a robust link between motor activity, including both whole-body and upper-limbs movements, and time perception. However, a full comprehension of the nature of this interaction, as well as the involved brain mechanism, is far from being achieved. Most of the previous studies have just measured the effect of motor activity in either one range (milliseconds) or the other (seconds). Moreover, there is a huge variability in the methods used to measure time performance (e.g., discrimination, reproduction, generalization) as well as in the kind of motor activity investigated (e.g., running or cycling) and, in particular, the exercise intensity level (moderate or vigorous). Finally, most of the previous studies mainly cope with visual stimuli leaving the effect of physical exercise on audition almost completely neglected. The aim of the current study was to employ a validated physical activity paradigm (Petrizzo et al., 2022) to directly compare its effects on different sensory modalities (visual and auditory) as well as duration range (sub − second and supra − second) in order to obtain a more generalized account of the influence of motor activity on duration perception. The prediction is clear: if time distortions induced by physical activity are the result of a unique, generalized timing mechanism, we might expect distortions to generalize across sensory modalities and time ranges.

## 2. Materials and methods

### 2.1. Power analysis

To estimate the sample size, we used as references the results obtained by (Lambourne, 2012; Kroger-Costa et al., 2013; Petrizzo et al., 2022). Like the current study, these studies employed a time generalization task to measure perceived durations during physical exercise and at rest. Specifically, Petrizzo et al. (2022) and Kroger-Costa et al. (2013) measured running-induced temporal biases for durations in the millisecond range (standard 600 ms) for visual and auditory stimuli. Lambourne (2012) instead measured cycling-induced biases for visual durations in the millisecond range (standards 300 and 600 ms). From these studies, we extracted and averaged Cohen’s d values for stats contrasting the results obtained at rest and during physical exercise. The between studies average Cohen’s d value was 0.88. Using software G*power (Faul et al., 2007), we then calculated the sample required for a (two-tailed) t-test against measuring the difference between two dependent means (physical exercise Vs resting), considering a significance level of α = 0.05 and power of (1-β) = 0.95. We found that a sample size of 19 participants would be needed.

### 2.2. Participants

A total of 33 participants with normal or corrected-to-normal vision and no auditory impairments participated in the study (5 authors, 28 naïve, 13 females, mean age 26.4 ± 4.96). Eighteen participants performed both the visual and auditory tasks, ten performed only the visual task, five only the auditory task. In sum, the visual task was completed by 29 participants while the auditory task was completed by 23 participants. Independently of the sensory modality, all participants performed the task for both temporal ranges (milliseconds and seconds). Participants’ sports habits were investigated by asking whether they practiced any sport and, if so, how many times per week over the past six months. Five participants reported no sporting activity. The others reported exercising two to three times a week with an average frequency of two/three days for one/two hours each time. The activities performed were heterogeneous: artistic gymnastics, weightlifting, tennis, dance, volleyball, boxing, running, ultimate frisbee, martial arts.

All participants provided written informed consent and a medical certificate for non-competitive physical activity. Each experimental condition lasted about 2h per participant. Due to the demanding nature of the experimental procedure, both in terms of physical effort and time commitment 15 participants were unable to perform both the visual and the auditory condition. The research was approved by the local ethics committee (*“Commissione per l’Etica della Ricerca”*, University of Florence, 7 July 2020, n.111).

### 2.3. Apparatus and stimuli

For each condition, participants were standing or running on a treadmill (JK Fitness Top Performa 186), in a dimly lit and quiet room at approximately 90 cm from a monitor (Telefunken Smart TV 43”). Auditory stimuli were delivered by Bluetooth high quality headphones (Sony WF-SP800N). Heart rate was continuously monitored via a Bluetooth connection via a Garmin Forerunner 55 smartwatch paired with an HRM-Dual Heartrate strap. Following our previous experiment (Petrizzo et al., 2022), in the visual version of the experiment, intervals were marked by the on and offset of a centrally displayed blue square (subtending an area of approximately 15°X15° at the viewing distance of 90 cm). In the auditory version of the experiment, stimuli consisted of pure tones with a frequency of 1,000 kHz and an intensity of 75 dB measured at the sound source. In all experiments, participants judged the duration of the test stimuli against a reference of either 0.4 s (sub-second range) or 2 s (seconds range) tested in separated sessions. The test durations were logarithmically spaced around the standards, with a constant difference between successive durations of approximately 25%. In detail, in the milliseconds range, test durations were: 0.2, 0.252, 0.318, 0.4, 0.504, 0.634, and 0.798 s while for the seconds range were: 1.002, 1.262, 1.589, 2, 2.518, 3.170, and 3.990 s. Stimuli were generated and presented with PsychToolbox 3 routines in Matlab 2016b (Brainard, 1997; Pelli, 1997; Kleiner et al., 2007).

### 2.4. Procedure

**FIGURE 1:**
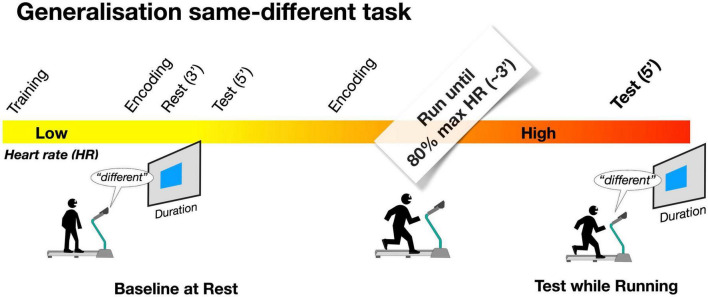
Schematic representation of the paradigm. After a training session, participants were presented with the reference stimulus five times (Encoding phase). After three minutes of rest, the baseline was measured, at rest (see methods for details). Then, after a second encoding phase, participants started running. After three minutes of running the timing task was repeated, this time while participants were running at 80% of their maximal heart rate.

Time perception was measured with a time generalization same-different task. A schematic representation of the procedure is depicted in Figure 1. The milliseconds and seconds range, as well as the visual and auditory modality, were tested in separate sessions. Each experiment included an initial training session. This session started with an “encoding phase” in which the reference stimulus was repeated sequentially five times with no response required. Then, in a block of seven trials, all possible durations of the test stimuli were randomly presented with participants required to judge the interval to be the same or different compared to the previously learned standard. In this phase response feedback was provided by a color change of the central fixation point (green for correct responses, red for mistakes). Every seven trials, the percentage of correct responses was calculated, and the training continued (in blocks of seven trials) until the percentage of 85% of correct responses was achieved. After the training, a new encoding phase started with the reference stimulus presented five times followed by a resting of 3 min. At the end of the resting phase participant started the baseline condition. This consisted of 54 test trials, with each test duration presented 6 times, and the standard duration 18 times. As for the training, participants had to report whether test duration was the “same” or “different” from the reference duration. Baseline was followed by another encoding phase, at the end of which the participant started the running phase. During the first 3 minutes of running no stimuli were presented and the treadmill speed was adjusted to make participants reach a pre-defined heart rate corresponding to the 80% of the maximum heart rate for his/her age following the formula: 208 – (0.7 * age); see (Tanaka et al., 2001). To reach and maintain the heart rate at the target value, the treadmill speed was continuously adjusted by the experimenter and all participants succeeded in reaching the target heart rate within 3 min. After this preparation phase, the timing task was repeated while running. This second test phase lasted about 4 min for the milliseconds range and about 5 min for seconds range, with the total running time of the block lasting 7 and 8 min, respectively. During the decoding phase, the treadmill speed was adjusted, if necessary, to keep participants heart rate around the target (80% of the maximum heart rate).

After the testing phase, the participants were allowed to take a break and rest, and when the heartbeat had returned to baseline levels (± 10 bpm) the whole procedure (apart from the training) was repeated in the same temporal order. At the end of the experimental session, each participant had completed two blocks per condition, for a total of 108 trials.

### 2.5. Running variables and heartbeat parameters

**FIGURE 2:**
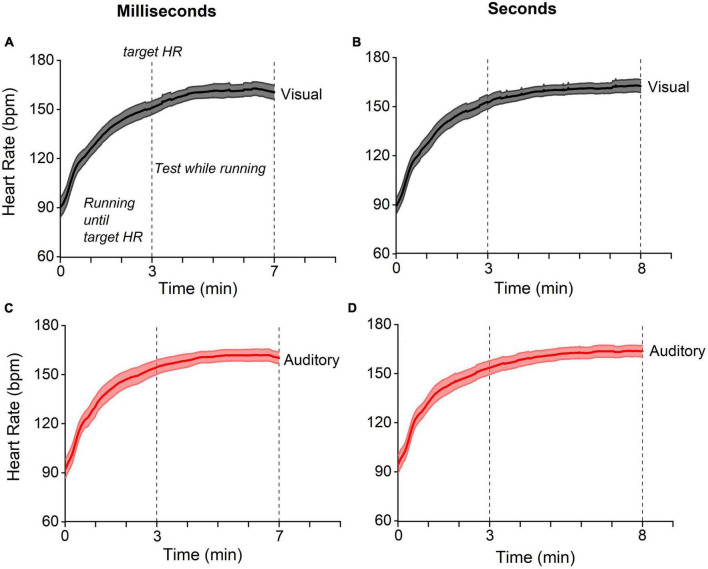
Heart-rate temporal trajectories. Temporal trajectories of heart rate for visual (gray) and auditory (red) experiments for stimuli in the ranges of milliseconds (panels on the left) and seconds (on the right). In all experiments **(A–D)**, the heart rate gradually reached the target value (see methods) within three minutes and remained stable for the subsequent four/five minutes of running (testing phase). Lines reports between participants average, the shaded areas report 95% CI.

Heart rate and running speed were calculated during running since the target heart rate had been reached, while the number of steps refers to the whole running period. The baseline heart rate was obtained with a 1-min recording at rest. This measurement was repeated each time before the test session. In two auditory running conditions (one relative to the sub-second and one for the supra-second range) the heart rate of one participant was not collected due to technical failure; that also occurred for two participants in the auditory baseline condition (second range). Running speed and steps number of one participant were not collected in the auditory-milliseconds-while-running and in the auditory-seconds-while-running conditions. Figure 2 reports the participants’ average heart rate for the visual and auditory tasks in both interval ranges, milliseconds, and seconds. Heart rate increased during the first 3’ and remained constant until the end of running phase.

### 2.6. Data analysis

The proportion of “same” responses were plotted as a function of test duration and fitted with a Gaussian function. The peak of the fits describing the data distributions reflects the point of subjective equality of test and reference (PSE): the duration of the test stimulus being perceived to be the same as the reference stimulus. From the best Gaussian fit we also retrieved a measure of sensory precision indexed by Weber Fraction (Eq. 1). The magnitude of the temporal distortions induced by running was measured as the standardized difference between the PSEs measured at rest and while running (Eq. 2). Similarly, as an index of heart rate (HR) acceleration, we measured the difference between HR measured at rest and while running. As described in the dedicated section, the database was unbalanced with only a proportion of the sample who performed both the visual and acoustic task. For this reason, to statistically quantify the effects on accuracy and precision the raw PSEs or WFs were analyzed with a linear mixed model ANOVA. PSEs or WFs were entered as dependent variable, modality (visual and auditory), duration range (milliseconds, seconds) and motor condition (resting and running) were entered as fixed effects. Participants were entered as a random effect. For all conditions PSEs and Wfs were normally distributed (Shapiro-Wilk, all *p* > 0.05).

Complementing the frequentist ANOVA, we also ran a series of Bayesian t-tests (two-tailed) contrasting the normalized effect against zero (no effect) or between temporal regimes. As not all participants completed the visual and auditory tasks but all of them completed, for a given sensory modality, the tasks with short and longer durations, we did not compare the normalized effects between visual and auditory modalities (information however provided by the ANOVA). Bayesian statistic was also reported for correlations (Pearson’s r, two-tailed) between the normalized effects across conditions as well as between normalized effects and HR acceleration. For the same reason, we only performed correlations within modalities and not between modalities. For these statistics we measured Bayes Factors, the ratio of the likelihood of the alternative to the null hypothesis and reported them as base 10 logarithms (LBF) (Jeffreys and Jeffreys, 1998; Lavine and Schervish, 1999; Jarosz and Wiley, 2014). By convention (Jarosz and Wiley, 2014) LBF from 0 to 0.47 is considered weak evidence for the alternative hypothesis, LBF > 0.47 is considered substantial evidence in favor of the alternative hypothesis and LBF < -0.47 substantial evidence for the null hypothesis. Absolute values greater than 1 are considered strong evidence, and greater than 2 definitive. Data were analyzed by JASP (Version 0.16.3) and Matlab software. Matlab was used to fit the timing task data with Gaussian functions to estimate PSEs and Wfs. JASP was used for all the other statistical tests.


(1) $$W\,f=10^{\sigma }-1$$

Where σ reflects the standard deviation of the Gaussian fit (on a log range) describing the proportion of “same” responses against test stimulus duration.


(2) $$N\,o\,r\,m\,a\,l\,i\,z\,e\,d\,e\,f\,f\,e\,c\,t=\frac{P\,S\,E\,\left(r\,u\,n\,n\,i\,n\,g\right)-P\,S\,E\,\left(r\,e\,s\,t\right)}{P\,S\,E\,\left(r\,u\,n\,n\,i\,n\,g\right)+P\,S\,E\,\left(r\,e\,s\,t\right)}$$

Where PSE running and PSE rest reflect the Point of Subjective Equality measured in the running or baseline (resting) condition, respectively.

## 3. Results

### 3.1. Perceived duration: Aggregate data

As described in the methods section, participants were asked to compare the duration of a series of visual or auditory stimuli to the duration of a previously visual or auditory reference stimulus (different sensory modalities investigated in separated sessions). Depending on the condition the reference could last 0.4 s (“milliseconds range”) or 2 s (“seconds range”) with test stimuli ranging from 0.2 to 0.8 s or from 1 to 4 s, respectively. All the conditions were tested in separate blocks with participants resting or running on a treadmill.

**FIGURE 3:**
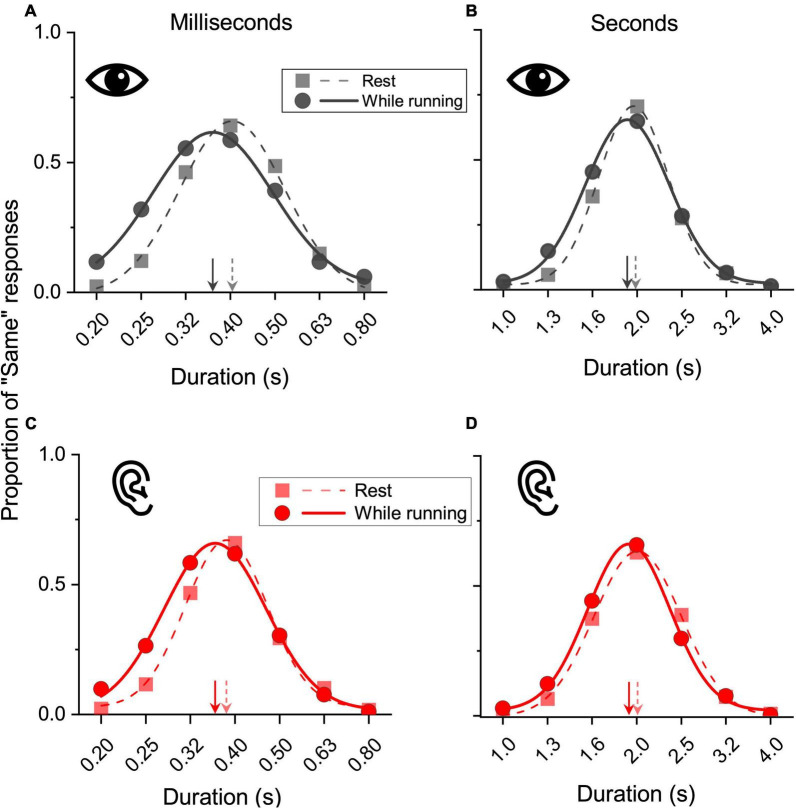
Results on aggregate data. Data for visual **(A,B)** and auditory **(C,D)** stimuli belonging to the subsecond (reference 400 ms: **A,C**) and second range (reference 2 s: **B,D**) obtained while resting (squares, dashed lines) or while running (circles, continuous lines). Test stimuli durations were plotted against the proportion of “same” responses and fitted with Gaussian functions. The peaks of the fits (arrows) correspond to the PSEs. A relative leftward shift corresponds to a duration overestimation of the test stimuli.

Figure 3 shows the results for the aggregate data. Panels A and B show the results obtained in the visual modality, for short and longer stimuli, respectively, while panels C and D show the results for auditory stimuli. On visual inspection, it is evident that all the curves obtained while running were shifted leftward relative to those measured while resting, indicating that duration was overestimated while running. In the resting conditions the peaks were all near to the physical reference duration (milliseconds range: 0.4 s and 0.38 s for vision and audition; seconds range: 1.9 s and 2 s for vision and audition). In the milliseconds range, while running, for both vision and audition a stimulus lasting 0.36 s was perceptually judged as equivalent to the 0.4 s reference, an overestimation of about 9%. Similar effects emerged in the seconds range. While running, both a visual and an auditory stimulus lasting 1.9 s were perceptually judged as equivalent to the 2 s reference, an overestimation of about 5%.

### 3.2. Perceived duration: Group analyses and individual data

**FIGURE 4:**
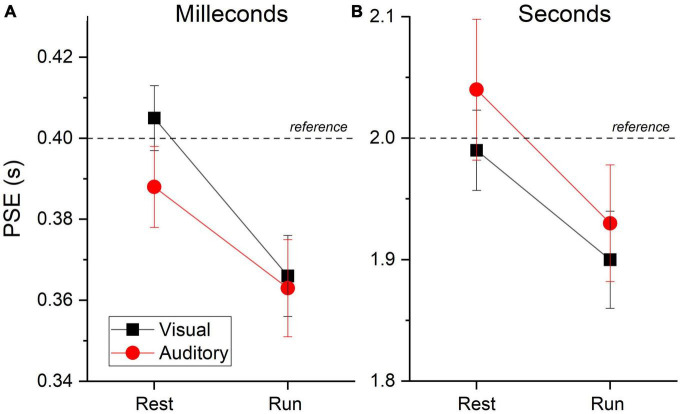
PSEs averages. Between subjects PSEs average for visual (black squares and lines) and auditory (red circles and lines) stimuli belonging to the milliseconds **(A)** and seconds **(B)** ranges divided by motor condition (resting and running). Dashed lines reports reference duration. Relatively lower values reflect duration overestimation of the test stimuli. Error bars are ± 1SEM.

With the same fitting procedure used for the aggregate data, we also analyzed the data separately for each participant and condition. For all the Gaussian fits on the individual data, an R^2^ higher than 0.7 was achieved. Figure 4 shows the between participants PSEs average for stimuli in the milliseconds (A) and seconds (B) range for visual and auditory stimuli. The results mirrored those obtained with aggregate data with almost veridical duration perception while resting (values around the reference line) and a clear duration overestimation (lower PSEs values) while running. A visual inspection confirms a similar pattern of results across sensory modalities and stimuli durations range (see Table 1 for PSEs descriptive statistics).

**TABLE 1:** Descriptive statistics (PSE).

Milliseconds							
		**N**	**Mean**	**St. err.**	**SD**	**Min**	**Max**
Visual	Rest	29	0.405	0.008	0.044	0.31	0.5
	Run	29	0.366	0.01	0.059	0.26	0.48
Auditory	Rest	23	0.388	0.01	0.047	0.3	0.5
	Run	23	0.363	0.012	0.056	0.25	0.47
**Seconds**							
		**N**	**Mean**	**St. err.**	**SD**	**Min**	**Max**
Visual	Rest	29	1.99	0.033	0.176	1.73	2.35
	Run	29	1.9	0.04	0.228	1.46	2.5
Auditory	Rest	23	2.04	0.058	0.279	1.66	2.84
	Run	23	1.93	0.048	0.231	1.47	2.32

PSEs, point of subjective equality; N, number of observations; Mean, between participant’s average; St. err., standard error of the mean; SD, standard deviation; Min, minimum; Max, maximum.

A linear mixed ANOVA, together with an obvious effect of duration range (*F*(1,169.56) = 6061, *p* < 0.001) indicating that PSEs scales with stimuli duration, revealed a main effect of motor condition, confirming lower PSEs values (duration overestimation) while running compared to resting (*F*(1,169.56) = 9.87, *p* = 0.002). Crucially all the interactions were not statistically significant (see Table 2) indicating a similar effect of running on PSEs across sensory modalities and stimuli duration range. The same results were replicated when the five authors where removed from the data set (see Table 4 of the Supplementary materials).

**TABLE 2:** Mixed ANOVA on PSEs, summary table.

Parameter	df	F	p
Modality	1, 197.95	0.234	0.629
Range	1, 169.56	6061.650	<0.001
Motor	1, 169.56	9.879	0.002
Modality*Range	1, 169.56	1.631	0.203
Modality*Motor	1, 169.56	0.006	0.940
Range*Motor	1, 169.56	2.532	0.113
Modality*Range*Motor	1, 169.56	0.188	0.665

PSEs, point of subjective equality.

**FIGURE 5:**
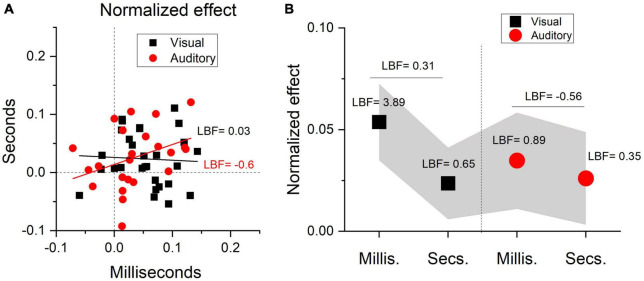
Normalized effects. **(A)** Single subjects’ data reporting the running effect measured in the seconds range against that measured in the milliseconds range divided by stimuli sensory modality (auditory: red circles, visual: black squares). Positive values indicate a temporal overestimation.. Continuous lines report best linear fits. **(B)** Between participants average effect as a function of experimental conditions (Auditory: red circles, Visual: black squares, Millis.: milleconds range, Secs: seconds range). Shaded area report 95% CI.

To better visualize the effects induced by running we calculated, separately for each participant, a standardized index of the effect’s magnitude (see Eqn. 2). Figure 5A shows the results obtained within the seconds range against those found in the milliseconds range, for auditory (red) and visual (black) stimuli. Despite a large interindividual variability, most of the data points fall in the positive quadrant, confirming a temporal overestimation induced by the running phase. The data points for visual and auditory stimuli were largely overlapped, confirming similar effects across sensory modalities. The average effects, together with associated 95% CI (shaded area) are depicted in Figure 5B.

Complementing the frequentist linear mixed ANOVA, we also ran a series of Bayesian t-tests contrasting the normalized effects against zero (no effect). For visual stimuli in the seconds range, the results provided substantial (LBF = 0.65) evidence in favor of H1. For visual stimuli in the milliseconds range the evidence for H1 was decisive (LBF = 3.89). Regarding auditory stimuli, the results revealed substantial evidence for H1 in the case of short durations (LBF = 0.89) and weaker (LBF = 0.35) evidence for H1 in the case of longer durations.

As described in the methods, not all the participants performed both the acoustic and the visual tasks. However, within the two modalities, all participants performed the tasks for both stimuli in the milliseconds and seconds ranges. To further characterize the effects across temporal regimes, we compared and correlated the effects between the milliseconds and seconds ranges, separately for visual and auditory stimuli. For auditory stimuli the results suggested substantial evidence in favor of H0 (no difference, LBF = − 0.56). For visual stimuli the results suggested weak evidence for H1 (LBF = 0.31). To investigate the links between the effects, we thus run two correlations, one for the visual and one for auditory stimuli, contrasting the effects measured for short (milliseconds) and longer (seconds) stimuli. If the effects for the two temporal regimes originate from a unique mechanism dedicated for both, we expect positive correlations. Contrarily to the prediction, the results showed no evidence for correlations, for both visual (*r* = − 0.078, LBF = − 0.6) and auditory (r = 0.37, LBF = 0.03) stimuli (Figure 5 A).

Overall, these series of Bayesian checks on the normalized effects, confirmed the results provided by the frequentist linear mixed model, suggesting that running had a similar effect on both short and longer durations within visual and auditory modalities.

### 3.3. Correlations with heart rate

As previous results obtained with both running (Petrizzo et al., 2022) and cycling (Tonelli et al., 2022) procedures suggested an independence between timing biases and heart rate acceleration induced by exercise, we also ran a series of correlations between the perceptual biases induced by the running phase and heart rate modulations. As an index of heart rate modulation induced by the running phase we calculated, separately for each participant and condition, the difference between the heart rate measured at rest (see methods) and the average heart rate measured while running (without considering the first three minutes needed to reach the HR threshold, see methods). The average heart rate modulation for the visual conditions were: 71 and 71.4 beats per minute for stimuli in the milliseconds and seconds range, respectively. The average heart rate modulation for the auditory conditions were: 62.2 per minute for both temporal ranges. Importantly for the correlational analysis, across all the conditions, there was a substantial interindividual variability, with heart rate modulation varying between a minimum of around 50 to a maximum of 88 beats per minute (descriptive statistics are reported in Table 3 of the Supplementary materials). The results showed substantial evidence for no correlation between heart rate modulation and effect’s magnitude for all four conditions (all *p* > 0.46, max LBF = − 0.49, see and Table 3).

**TABLE 3:** Correlations between the effect’s magnitude and heart rate modulation.

Condition	Pearson’s r	p	LBF
Visual seconds	-0.14	0.469	-0.53
Visual milliseconds	0.097	0.615	-0.58
Auditory seconds	-0.14	0.55	-0.49
Auditory milliseconds	-0.116	0.61	-0.52

Heart rate modulation = (Heart rate measured while running − Heart rate measured at rest); LBF, base ten log Bayesian factor.

**TABLE 4:** Descriptive statistics (Weber Fraction).

Milliseconds							
		**N**	**Mean**	**St. err.**	**SD**	**Min**	**Max**
Visual	Rest	29	0.276	0.012	0.065	0.15	0.4
	Run	29	0.32	0.017	0.093	0.17	0.54
Auditory	Rest	23	0.209	0.01	0.047	0.14	0.29
	Run	23	0.279	0.016	0.076	0.17	0.41
**Seconds**							
		**N**	**Mean**	**St. err.**	**SD**	**Min**	**Max**
Visual	Rest	29	0.187	0.012	0.065	0.08	0.33
	Run	29	0.222	0.016	0.088	0.09	0.42
Auditory	Rest	23	0.19	0.01	0.05	0.1	0.29
	Run	23	0.22	0.018	0.085	0.08	0.4

N, number of observations; Mean, between participant’s average; St. err., standard error of the mean; SD, standard deviation; Min, minimum; Max, maximum.

### 3.4. Sensory precision

**FIGURE 6:**
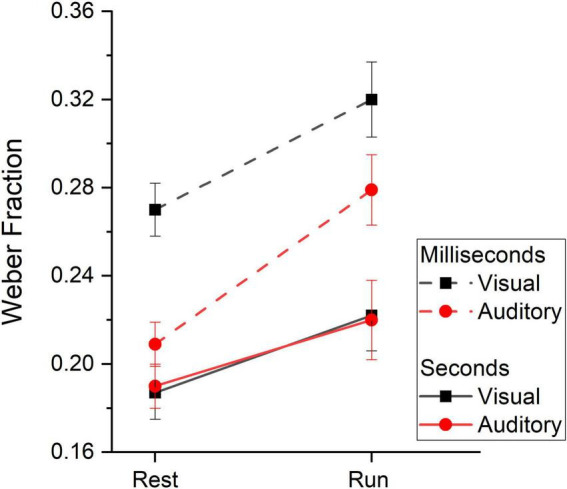
Weber Fractions. Between subjects’ average Weber Fraction measured at rest and while running divided by duration range (subsecond: dashed lines, second: continuous lines) and stimuli sensory modality (vision: black squares and lines, audition: red circles and lines). Error bars are ± 1SEM.

To assess whether running yielded distortion on the precision of sensory estimates across sensory modalities and duration ranges, we analyzed Weber Fractions (Wfs, see Table 4 for descriptive statistics). Figure 6 shows between subjects’ average Wfs measured while resting and while running. On inspection it is evident that Wfs in the milliseconds range were higher (lower precision) compared to those measured in the seconds range, regardless of the sensory modality. Collapsing together visual and auditory data, Wfs were on average 0.27 and 0.2 for the milliseconds and seconds ranges respectively. Another result emerging from the data reported in Figure 6 is that, in the two temporal ranges, Wf varies differently between sensory modalities. In the milliseconds range, Wfs were lower for the auditory modality than that obtained in the visual modality while, for longer durations (seconds range), visual and auditory Wfs were identical. This latter result was independent from the motor condition and held true when time perception was measured while resting as well as during running. Finally, for both vision and audition and for both temporal ranges, Wfs were higher while running compared to the resting condition (Wfs = 0.26 and 0.21 for the running and resting conditions, respectively).

A linear mixed ANOVA (Table 5) provided a statistically significant effect of range (*F*(1,166.26) = 50.93, *p* < 0.001), indicating higher precision for the seconds range than the millisecond range. The motor condition also yielded a significant result (*F*(1,166.26) = 25.99, *p* < 0.001) confirming higher precision at rest compared to during running as well as a statistically significant effect of modality (*F*(1,196.97) = 4.877, *p* = 0.02) indicating higher precision for auditory stimuli. Crucially, the modality*range interaction was also statistically significant (F(1,196.97) = 10.24, *p* = 0.002) confirming lower precision in the visual modality, but only in the milliseconds range. All the other interactions were not statistically significant, confirming that visual and auditory Wfs varied similarly across motor conditions (modality*motor = *F*(1,196.97) = 0.43, *p* = 0.51), the effect of running was similar across temporal ranges (range*motor = *F*(1,196.97) = 1.48, *p* = 0.22) and that the effect of running on precision was similar across temporal ranges and sensory modalities (modality*range*motor = *F*(1,196.97) = 0.49, *p* = 0.48). The same results were replicated when the five authors were removed from the dataset (see Table 5 of the Supplementary materials).

**TABLE 5:** Mixed ANOVA summary table (Weber Fraction).

Parameter	df	F	p
Modality	1, 196.97	4.877	0.028
Range	1, 166.26	50.931	-0.001
Motor	1, 166.26	25.990	-0.001
Modality*Range	1, 166.26	10.241	0.002
Modality*Motor	1, 166.26	0.439	0.509
Range*Motor	1, 166.26	1.481	0.225
Modality*Range*Motor	1, 166.26	0.494	0.483

## 4. Discussion

In the current study we measured the effect of running on time perception for short (milliseconds) and longer (seconds) durations for both visual and auditory stimuli. The results obtained with a standard generalization task (Lambourne, 2012; Petrizzo et al., 2022) confirmed previous studies showing that subjective time for visual short durations is expanded during a vigorous running phase. The results then expanded the previous findings by showing that the perceived time expansion also occurs for longer durations (in the seconds range) and regardless of stimuli sensory modality (visual or auditory).

It is worth mentioning that the current results on perceptual biases were partially different from those obtained in the only available study investigating the effect of physical exercise on auditory time perception. As mentioned in the introduction, Kroger-Costa et al. (2013) measured auditory time perception in the range of milliseconds (300-700 ms) during running, compared to a resting condition. Time perception was measured with a discrimination task in which participants were asked to classify durations as “long” or “short” in terms of their relative similarity to two previously learned anchors setting the minimum and the maximum of the stimuli range, as well as by a generalization task, like that used here. The results obtained with the discrimination task showed a significant time overestimation induced by running, like the current results. However, at odds with the present study, the generalization task did not provide any significant effects. While these results might indicate that a discrimination task could be more sensitive to measure timing biases during physical exercise, the deviation from our results might also stem from a combination of methodological differences. The first is related to the lower sample size recruited in the mentioned study (10 participants), compared to the current experiment (23 participants for the auditory task). In line with this possibility, while the effect for auditory stimuli in the subsecond range found here was on average reasonably robust, there was also a considerable inter-individual variability (see Figure 5A). A sample of 10 participants, given the level of variability, might not have been sufficient to reveal any significant effect in the Kroger-Costa et al.’s (2013) study. A second difference regards the running procedure. In the Kroger-Costa study the physical effort was not equalized between participants and a fixed treadmill speed equal to 7.2 Km/h was used for all participants. Here we equalized the physical effort across participants by defining for each participant a given heart rate value that was reached by dynamically adjusting the treadmill speed (80% of the maximal, see methods) during the running phase. This procedure resulted in an average running speed of about 10 Km/h, which was considerably higher compared to 7.2. Overall, the fixed treadmill speed procedure used by Kroger-Costa et al. (2013) could have made the physical activity effort not sufficiently strong (at least for some participants) to elicit a measurable effect on time perception, washing out the average effect. This partial discrepancy between studies calls for further investigation on the role of the aforesaid methodological differences.

It is of interest to compare the present data also with the study by Tonelli et al. (2022) investigating the effect of moderate cycling on visual time perception. The results, obtained with a reproduction paradigm, showed that while temporal distortions were qualitatively present across all the tested durations, they were statistically different from zero just in the milliseconds range (200 − 800 ms vs. 1.6 − 3.2 secs). In the current experiment, replicating previous evidence (Sayalı et al., 2018) we instead found that the effect for visual stimuli in the seconds range was clearly above zero (LBF = 0.65). Moreover, the ANOVA showed no interactions between motor conditions (rest, run) and stimuli regimes (milliseconds, seconds), confirming similar effects for short and longer durations. However, it should also be noted that the Bayesian analysis directly contrasting the visual effects between regimes, returned an LBF of 0.31 that, by convention, indicates a weak and non-decisive result, leaving open the possibility for smaller time distortions in the seconds regime. Partial discrepancies between the two studies might also suggest that different types of physical exercise and different experimental procedures to measure time perception (reproduction vs generalization tasks) are likely to yield different effects, probably tapping into different time mechanisms. Indeed, compared to cycling, running is a more complex motor routine, involving all four limbs and requiring a higher level of proprioception and balance. Moreover, during running the continuous up and down movement of the head might trigger a series of complex vestibulo-ocular movements aimed at stabilizing the eye relatively to the external world (Purves et al., 2001), these movements that are significantly mitigated during cycling. These and probably other factors might have contributed to the differences observed in the effects of running and cycling on time perception including the fact that, while the effect of cycling has been reported to last several minutes after the end of the exercise (Tonelli et al., 2022), the effect of running on time perception seems to fade out immediately after the running period (Petrizzo et al., 2022). With the current results, we cannot determine which factors underlie these differences, but these certainly highlight the need for studies directly comparing the effects yielded by different physical exercises on time perception.

Together with a rather a-specific effect of running on time, we also collected clear evidence for partially separate systems involved in the encoding of short/long durations and between stimuli modality (vision vs. audition). This result cannot be trivially accounted for in terms of a methodological weakness related to some of the participants not taking part to both experiments. Indeed, we overcame this issue by leveraging on Mixed Models capable to preserve statistical power also in cases of non-homogenous data sets. The claim for distinct time mechanisms for vision and audition is also in line with previous findings: sensory precision levels were higher for stimuli in the seconds range compared to stimuli belonging to the milliseconds range (Hayashi et al., 2014). Furthermore, as found by Rammsayer et al. (2015), the results obtained here showed higher sensory precision for auditory stimuli but only for short durations, in the milliseconds range. Overall, it is difficult to explain the results on sensory precision with a single mechanism encoding time across temporal ranges and sensory modalities, and the results are in line with the previously suggested possibility of a smooth transition from a sensory modalities specific timing mechanism encoding short durations, to a more sensory independent mechanism encoding longer stimuli (Rammsayer et al., 2015; Rammsayer and Pichelmann, 2018; Bratzke and Ulrich, 2019). Moreover, within both sensory modalities, the effects of running for stimuli in the milliseconds and seconds ranges were weakly or not at all correlated between each other, again in line with the idea of partially separate mechanisms for short and longer durations. Despite this, the temporal biases induced by running were qualitatively and quantitatively similar across conditions, indicating that these temporal mechanisms − whether separated or not − nevertheless make use of shared resources linked to the motor system.

Which factor (or factors) underlies the observed effects on time estimates? One possibility we can easily exclude is that sensory precision level was driving the effects. Indeed, running similarly affected the milliseconds and seconds ranges despite the fact that they had clearly different precision levels. Moreover, in the milliseconds range, while the effects were similar for vision and audition, the sensory precision level was much higher for auditory stimuli. The independence between sensory precision and timing contextual effects was not granted. Indeed, it has been previously shown that auditory time perception, compared to visual, is much less susceptible to contextual factors such as the well-known central tendency effect (a general perceptual phenomenon dragging the current perception towards the average of the tested range) (Cicchini et al., 2012). The robustness of the acoustic timing system to this contextual effect has been linked to its higher sensory precision, compared to the visual modality. Another factor we can reasonably exclude is heart rate modulation. The results, confirming previous evidence (Petrizzo et al., 2022; Tonelli et al., 2022) demonstrated that the heart rate acceleration from the resting state to the maximal effort during running was not predictive of the effect magnitude, across all the experimental conditions. Heart rate level has long been considered a reliable indicator of arousal (Thayer, 1970), both of which might influence the speed of the internal clock. Although arousal might be not the driving factor, it is in line with previous results showing that, while time perception is distorted during physical exercise, estimates for other visual properties such as numerosity and spatial separation remain veridical (Petrizzo et al., 2022; Tonelli et al., 2022). Moreover, with the very same methods used here, we recently found that the effect of running on time perception vanished soon after the running phase while heart rate and, thus likely, arousal were still well above the baseline level (Petrizzo et al., 2022). This last result also makes unlikely explanations based on changes in the release of hormones or neurotransmitters, as these also take time to fully reuptake.

It has been previously suggested that physical exercise might change time perception through a generic deprivation of the cognitive resources that are allocable to the timing task (Behm and Carter, 2020). According to this idea, running would act as a distractor task, dragging attention and cognitive resources away from the timing task thus modifying temporal encoding and hampering accuracy and precision. In line with this hypothesis, the current results showed that sensory precision worsens during running, compared to the resting state. Although it could be argued that running represents a rather automatized motor routine, this might not be true in our case where running was performed on a treadmill, a condition not entirely familiar for most participants. It should be noted that this (probably simplistic) explanation is in line with the lifespan of the effect induced by running. As soon as the running ends and thus attentional resources were released, the distortion on time perception vanished (Petrizzo et al., 2022). The idea of a generic attentional deprivation induced by running could also explain why the effect generalizes to stimuli of different durations and sensory modalities. This explanation might appear at odds with the fact that running does not interfere with visual number perception, however, there is much evidence suggesting that numerosity perception is fairly attentional free (Burr et al., 2010; Anobile et al., 2012, 2020b). Despite these indications, it is worth to be noted that according to the attentional gate model (Zakay and Block, 1997) we should assume that running, as a distractor, would results in a loss of temporal information and therefore in an underestimation of duration, which is opposite to the observed pattern of result. On the other hand, running could induce a generalized increase of attention to the timing task, resulting in an overestimation. In line with this, it has been suggested that high intensity physical exercise increases awareness (Edwards and Polman, 2013), which would, in turn, increase to the amount of attention given to duration. Moreover, previous reports supported the idea that higher attention to time usually induce overestimation biases (Behm and Carter, 2020; Martinelli and Droit-Volet, 2022). This idea while not against the attentional gate model, is however difficult to reconcile with the decrement of sensory precision observed while running, compared to the resting condition (paying more attention would increase precision during running). Obviously, all these hypotheses are at present speculations and would need future ad-hoc experiments to be tested.

## 5. Conclusion

In the current study we found that perceived duration of short (milliseconds) and longer (seconds) visual as well as auditory stimuli is expanded during running. Despite this a-specific effect of running on accuracy, the results on sensory precision are in line with the existence of different mechanisms for short and longer stimuli as well as between sensory modalities. While the factors underlying the effect of running on time perception remains largely unknown, the current results suggest that a full comprehension of the interplay between action and time perception can only be achieved by multifaced approaches involving different experimental paradigms, stimuli sensory modality, physical efforts and temporal regimes.

## Data availability statement

The datasets presented in this study can be found in online repositories. The names of the repository/repositories and accession number(s) can be found below: https://doi.org/10.5281/zenodo.7525564.

## Ethics statement

The studies involving human participants were reviewed and approved by the Local Ethics Committee (“Commissione per l’Etica della Ricerca”, University of Florence, 7 July 2020, n.111). The patients/participants provided their written informed consent to participate in this study.

## Author contributions

IP, GA, and RA conceived and designed the experiments. EC and TB performed the experiments and analyzed the data. IP and GA analyzed the data and wrote the first draft of the manuscript. RA reviewed and critiqued the manuscript. All authors read and approved the final manuscript.
