# Supplementary material for: Similar effect of running on visual and auditory time perception in the ranges of milliseconds and seconds
Source: Front Psychol. 2023 Mar 31;14:1146675. doi: 10.3389/fpsyg.2023.1146675 (PMC10102424; doi:10.3389/fpsyg.2023.1146675)
Supplement: Supplementary file 1 [file Data_Sheet_1.docx]

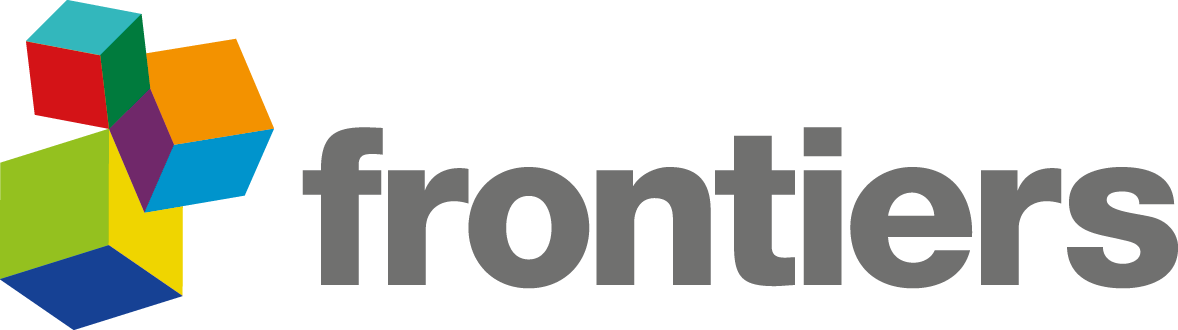


Supplementary Materials

| **Table 1. Running parameters** | | | |
| --- | --- | --- | --- |
| **Condition** | **Heart rate (Bpm)** | **Running speed (Km/h)** | **Steps per seconds (Hz)** |
| **Visual** | | | |
| **Milliseconds**  **Baseline** | 89±0.17 | n.a. | n.a. |
| **Milliseconds**  **While running** | 159.65±0.19 | 9.9±0.22 | 3.69 |
| **Seconds**  **Baseline** | 89.35±0.05 | n.a. | n.a. |
| **Seconds**  **While running** | 159±0.15 | 9.5±0.19 | 3.92 |
| **Auditory** | | | |
| **Milliseconds**  **Baseline** | 92.9±0.06 | n.a. | n.a. |
| **Milliseconds**  **While running** | 160±0.14 | 9.7±0.31 | 3.4 |
| **Seconds**  **Baseline** | 92.6±0.08 | n.a. | n.a. |
|  |  |  |  |
| **Seconds**  **While running** | 161±0.17 | 8±0.13 | 3.68 |
| Bpm= beats per minute; Km/h= kilometres per hour  Numbers reports between participants average, ± 1 s.e.m.  n.a.= not available | | | |

**Table 2. Descriptive statistics of Gaussian fits R^2^**

|  | | | | | |
| --- | --- | --- | --- | --- | --- |
| **Milliseconds** | | | | | |
|  |  | N | mean | SD | min |
| **Visual** | Rest | 29 | 0.95 | 0.055 | 0.74 |
|  | Run | 29 | 0.93 | 0.07 | 0.73 |
| **Auditory** | Rest | 23 | 0.97 | 0.03 | 0.85 |
|  | Run | 23 | 0.95 | 0.06 | 0.7 |
| **Seconds** | | | | | |
|  |  | N | mean | SD | min |
| **Visual** | Rest | 29 | 0.96 | 0.04 | 0.83 |
|  | Run | 29 | 0.96 | 0.04 | 0.82 |
| **Auditory** | Rest | 23 | 0.97 | 0.03 | 0.88 |
|  | Run | 23 | 0.95 | 0.06 | 0.72 |
| N= number of observations; Mean = between participant’s average; SD= standard deviation; Min=minimum | | | | | |

**Table 3. Heart-rate modulation descriptive statistics**

| **Condition** | **Mean** | **SD** | **Min** | **Max** |
| --- | --- | --- | --- | --- |
| **Visual Seconds** | 71.4 | 8.55 | 49 | 86 |
| **Visual Milliseconds** | 70.9 | 11 | 51 | 88 |
| **Auditory Seconds** | 66.2 | 7.8 | 53 | 78.2 |
| **Auditory Milliseconds** | 66.2 | 7.9 | 53 | 80 |
| Mean = between participant’s average; SD= standard deviation; Min= minimum; Max= maximum;  Numbers reports beats per minute | | | | |

**Table 4. ANOVA Summary (PSEs) only naïve subjects**

| **Effect** | **df** | **F** | **p** |
| --- | --- | --- | --- |
| Mod | 1, 160.00 | 0.451 | 0.503 |
| Range | 1, 134.78 | 4495.459 | <.001 |
| Motor | 1, 134.78 | 5.119 | 0.025 |
| Mod ✻  Range | 1, 134.78 | 1.286 | 0.259 |
| Mod ✻  Motor | 1, 134.78 | 0.069 | 0.793 |
| Range ✻  Motor | 1, 134.78 | 1.189 | 0.277 |
| Mod ✻  Range ✻  Motor | 1, 134.78 | 0.026 | 0.873 |
| PSEs=Point of Subjective Equality | | | |

**Table 5. ANOVA Summary (Weber Fractions) only naïve subjects**

| **Effect** | **df** | **F** | **p** |
| --- | --- | --- | --- |
| Mod | 1, 158.80 | 12.186 | < .001 |
| Range | 1, 131.93 | 67.290 | < .001 |
| Motor | 1, 131.93 | 21.788 | < .001 |
| Mod ✻  Range | 1, 131.93 | 11.444 | < .001 |
| Mod ✻  Motor | 1, 131.93 | 0.626 | 0.430 |
| Range ✻  Motor | 1, 131.93 | 1.559 | 0.214 |
| Mod ✻  Range ✻  Motor | 1, 131.93 | 0.304 | 0.582 |
